# Pneumonia

**Published:** 1904-04

**Authors:** 


### EDITORIAL NOTES AND COMMENTS.

The Business office of THE JOURNAL-RECORD is No. 1007-1008 Century Building.

The Editorial office is 318-319-320 The Prudential.

Address all Business communications to Dr. M. B. Hutchins, Mgr.

Make remittances payable to THE ATLANTA JOURNAL-RECORD OF MEDICINE.

On matters pertaining to the Editorial and Original Communications address Dr. Bernard Wolff, Atlanta.

Reprints of original articles will be furnished at cost price. Requests for the same should always be made *on the manuscript.*

We will present, post-paid, on request, to each contributor of an original article, twenty (20) marked copies of THE JOURNAL-RECORD containing such article.

### PNEUMONIA.

THE following is taken from the *Medical Standard* and is timely:

Although this subject was discussed, editorially, in the January number of the *Medical Standard*, no excuse seems necessary for bringing it up again. The increasing number of deaths from this cause makes it the dominant topic during the winter season, and thus it must remain until pneumonia becomes less prevalent and less deadly.

In the editorial just mentioned and in previous editorial expressions, the *Standard* has preached the wisdom of taking an optimistic view of the pneumonia problem. It is a problem to be sure; but that the disease is not amenable to medicinal treatment, as publicty stated by a distinguished Chicago physician not long ago, is, in our belief, not true. How much can be done in its treatment when this has a logical basis is shown by the experience of Dr. De Lancey Rochester, of Buffalo, who reports, in the *Medical News* of February 13, further trials of the method outlined by him some time ago, in conjunction with Dr. Charles G. Stockton. This was reported, at the time, in the *Standard.*

Dr. Rochester's ideas concerning the indications for treatment square, in the main, with those of the editor. They are: (1) To relieve the toxemia; (2) to prevent failure of the heart; (3) to meet complications as they arise. To accomplish the first of these ends Dr. Rochester stimulates the skin and bowels to carry off the constantly accumulating poisons. The bowels are kept clean and frequent liquid stools secured with daily doses of Epsom salts, following calomel at the outset of the disease. Free sweating is induced by hot mustard foot baths, given at frequent intervals, the patient being warmly covered with blankets. These baths, in connection with stimulation to maintain the action of the heart, are considered the most important of the therapeutic measures. Not only do they play an important eliminative part, but by dilating the capillaries they equalize the circulation and relieve the work of the heart. To maintain the work of the heart Dr. Rochester depends mainly upon strychnin, commenced early and given in doses sufficiently large. Next to strychnin he places alcohol. When there is evidence of failure of the right heart he bleeds. Locally he uses leeches and cups—wet and dry.

It will be observed that the essential features of this treatment are the eliminative ones, aimed at the toxemia—the continual flushing of the bowel and the diuresis induced by the mustard baths. These, as already stated, have a second and perhaps not less important effect, in that, by dilating the peripheral arterioles they dissipate the pulmonary stasis which endangers cardiac integrity. This treatment, therefore, seems to have a sound logical basis.

What are the results? Two years ago 168 cases treated by this method were reported, with twenty-one deaths. Of these, eleven were, to all intents and purposes, doomed when treatment was commenced. Excluding these the death-rate in this series was 6.33 per cent. Dr. Rochester now adds forty-two more cases, with four deaths, making a total of 210 cases treated by the eliminative method. Every case coming under observation, both in hospital and private practice, was included, among them being cases complicated with heart disease, nephritis, typhoid fever, delirium tremens, extreme old age (two patients were eighty-nine years old), etc. At least five had been sick a week before admission to the hospital, and several were practically moribund when treatment was commenced. The average mortality, including all these cases, was 11.4 per cent. Excluding the desperate, it was but seven per cent.; while if those over fifty were also deducted the death-rate would be but 2.5 per cent.

Compare these figures with those given by most authors. The hospital death-rate usually given is about twenty-five per cent., and that of cases in private practice is estimated at fifteen per cent. Wells collected over 200,000 cases having a mortality of eighteen per cent. The experience of Dr. Rochester should give the lie to the statement that pneumonia is not amenable to treatment. The course of pneumonia *can* be modified when the physician grasps firmly the indications to be met and, with a clear comprehension of the individual needs of each case, goes into the fight to win. A *laissez-faire* method of treating pneumonia never was very successful and never will be.

Whether the use of vasomotor depressants will yield equally good results we do not know, but they aim at practically the same end as the hot bath, and it seems reasonable to believe that they might be a valuable addition to the treatment outlined—if not carried to the point of systemic or cardiac depression.

If there is any moral to these facts, it is the necessity of a revival of faith in therapy. The pendulum is swinging back from the unfaith of the last decade to a rational and scientific therapeutics. Don't let your patients think that the doctor can do nothing for them in their struggle with death.

